# Management of Combined Therapy (Ceritinib, *A. cinnamomea*, *G. lucidum*, and Photobiomodulation) in Advanced Non-Small-Cell Lung Cancer: A Case Report

**DOI:** 10.3390/life12060862

**Published:** 2022-06-09

**Authors:** Chuan-Tsung Su, Jih-Huah Wu

**Affiliations:** 1 Department of Healthcare Information and Management, Ming Chuan University, Taoyuan 33348, Taiwan; ctsu@mail.mcu.edu.tw; 2 Department of Biomedical Engineering, Ming Chuan University, Taoyuan 33348, Taiwan

**Keywords:** non-small-cell lung cancer, photobiomodulation, *Antrodia* *cinnamomea*, *Ganoderma lucidum*, lung adenocarcinoma

## Abstract

The 5-year survival rate of non-small-cell lung cancer (NSCLC) is still low (<21%) despite recent improvements. Since conventional therapies have a lot of side effects, combined therapy is strongly recommended. Here, we report a patient with advanced NSCLC who received combined therapy, including ceritinib, photobiomodulation (PBM), ACGL (*Antrodia cinnamomea* (*A. cinnamomea*), and *Ganoderma lucidum* (*G. lucidum*)). Based on combined therapy, suitable doses of *A. cinnamomea*, *G. lucidum*, and PBM are important for tumor inhibition. This case report presents clinical evidence on the efficacy of combined therapy in advanced NSCLC patients, including computed tomography (CT) scan, magnetic resonance imaging (MRI), carcinoembryonic antigen (CEA), and blood tests. The effective inhibition of human lung adenocarcinoma cells is demonstrated. Our case highlights important considerations for PBM and ACGL applications in NSCLC patients, the side effects of ceritinib, and long-term health maintenance.

## 1. Introduction

Non-small-cell lung cancer (NSCLC) is one of the leading causes of death worldwide, imposing grievous challenges for patients and clinicians. In NSCLC, multiple driver oncogenes have been identified, including epidermal growth factor receptor [1], anaplastic lymphoma kinase (ALK) [2], Kirsten rat sarcoma viral oncogene homolog [3], c-ros oncogene 1 [4], v-Raf murine sarcoma viral oncogene homolog B1 [5], erb-b2 receptor tyrosine kinase 2 gene [6], rearranged during transfection [7], and c-mesenchymal-epithelial transition factor [8]. In the United States, approximately 85% of lung cancer cases are NSCLC patients [9].

Lung adenocarcinoma is the major subtype of lung cancer and approximately two-thirds of patients have locally advanced or metastatic disease [10]. Patients usually receive conventional therapies (i.e., chemotherapy or radiation therapy). Henk and Ray reported that the mean total cost of treatment for more than 300 patients with advanced NSCLC ranged from USD 19,182 to USD 167,847 and USD 35,737 to USD 135,364 for first-line and second-line management, respectively [11]. Despite the fact that cancer therapy has been improved, the 5-year survival rate of lung cancer remains at a very low level (<21%) in the last four decades [12]. Thus, a suitable combined therapy in lung cancer treatment is necessary.

A suitable dose of photobiomodulation (PBM) can slow down tumor growth and prolong the life span of mice [13]. The immunoglobulin activity (IgA, IgM, and IgG) in 60 oncologic patients can be increased with PBM in the tumoural area or lymph nodes [14]. The ratio of IgA and IgG can be increased for nearly 1.86-fold and 6.33-fold, respectively, by external irradiation with an 890 nm laser on the second day. In addition, the ratio of IgM can be increased nearly four-fold on the fifth day. Recently, PBM has been used for managing chemoradiotheray in head and neck cancer patients [15,16]. PBM with adequate radiation time can induce apoptosis in human lung adenocarcinoma cells in cell culture [17]. Therefore, a suitable PBM therapy can be used to treat cancer.

Polysaccharide has been used as an adjunctive therapeutic drug for the side effects during cancer treatment [18]. *G. lucidum* is an oriental fungus which has been widely used for promoting health. A highly antitumor activity from the polysaccharides of the fruiting body of *G. lucidum* was found in an animal model, which is mainly activated by the branched (1→3)–β–D–glucans [19,20]. Previous studies report that the biologically active polysaccharides from *G. lucidum* show high antitumor activity while enhancing the host’s immune response [20,21,22]. In addition, *A. cinnamomea* also shows antitumor activity that promotes a Th1-dominant state and natural killer (NK) cell activities through its polysaccharide components [23]. The triterpenoids from *G. lucidum* show a cytotoxicity-based carcinostatic effect on hepatoma cells in vitro [20]. The triterpenoids profile of *A. cinnamomea* fruiting bodies is richer than that of mycelia [24], and dish-cultured *A. cinnamomea* caused the tumor to shrink substantially for one small-cell lung cancer patient. The patient survived for 32 months without relapse after a 6-month treatment. In the present study, a combined therapy was administered to a lung adenocarcinoma patient in stage IVa.

## 2. Case Presentation

**Figure 1:**
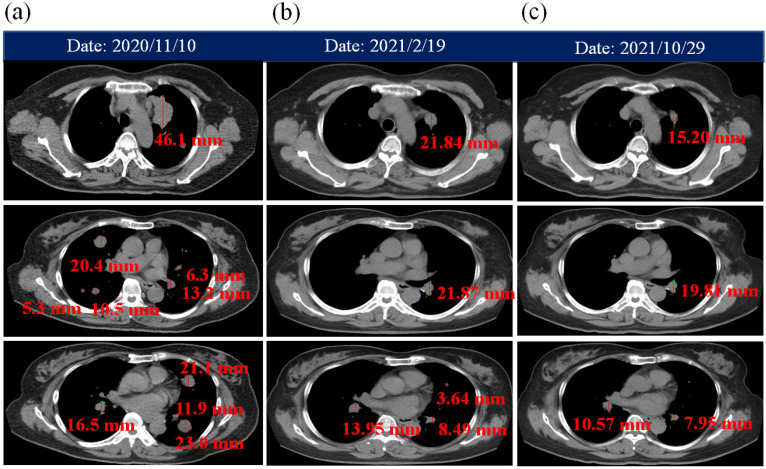
(**a**) Multiple pulmonary metastases of bilateral lung by chest CT. The CT scans of the patient whom received (**b**) 2 and (**c**) 10 months of combined therapy.

**Figure 2:**
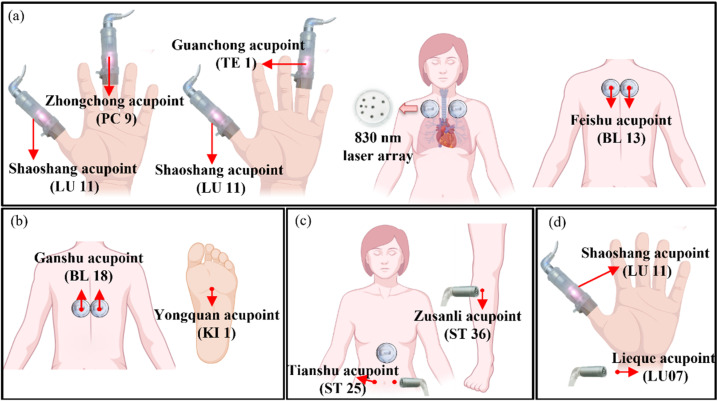
Schematics for the position of 830 nm laser on NSCLC patients. (**a**) Shaoshang (LU 11), Zhongchong (PC 9), Guanchong (TE 1), apex of lung and Feishu (BL 13) acupoints. The abnormal of ASL, ALT, and BUN levels can be modulated by 830 nm laser radiated on (**b**) Ganshu (BL 18) and Yongquan (KI 1) acupoints. (**c**) Tianshu (ST 25) and Zusanli (ST 36) acupoints and stomach area can be used for severe diarrhea treatment. (**d**) Shaoshang (LU 11) and Lieque (LU 07) acupoints can be used for severe dizziness treatment (The acupoints are marked by red arrows). Illustration created with BioRender.com (accessed on 8 April 2022).

A 60-year-old Asian woman, with a history of cough for 1 year, was admitted to a hospital in November 2020. Computed tomography (CT) scan (Figure 1a) was used to evaluate the adenocarcinoma in situ and multiple pulmonary metastases. The brain metastases in NSCLC were checked by magnetic resonance imaging (MRI). The size of the left upper lung cancer tissue was larger than 5 cm (5.49 × 3.04 × 4.61 cm^3^). After guided needle biopsy of the primary lung tumor, the woman was diagnosed with lung adenocarcinoma in clinical stage IVa NSCLC. She was treated with combined therapies, including PBM therapy, targeted therapy (ceritinib), ACGL (*A.* *cinnamomea* (50%), and *G. lucidum* (50%) at 450 mg ± 10%/capsule, manufactured by Well Shine Biothchnology Development Co., Ltd., Taipei, Taiwan). Three capsules of ceritinib per day (150 mg/capsule) and the oral administration of ACGL were prescribed. The dose of ACGL was increased to 12 capsules per day in August 2021. In addition, Multi-channel Laser Therapy System (Model: ID 310; wavelength: 830 nm and 650 nm; operation frequency: 10 Hz; 50% duty cycle; Jin-Ciang Technology Co., Ltd., Taiwan) was used to radiate on the apex of the lung and acupoints. The therapeutic protocol of the 830 nm laser was administered in four sequences once a day, each sequence for 10 min: (1) the Shaoshang (LU 11) and Zhongchong (PC 9) acupoints; (2) the Shaoshang (LU 11) and Guanchong (TE 1) acupoints; (3) the apex of the lung; and (4) the Feishu (BL 13) acupoint. The radiation position of the 830 nm laser is shown in Figure 2a. For transthoracic PBM therapy, the 830 nm array laser (7 laser diodes) was used to radiate on the chest (the apex of the lung) and the back area (BL 13 acupoint) for 10 min once a day.

**Figure 3:**
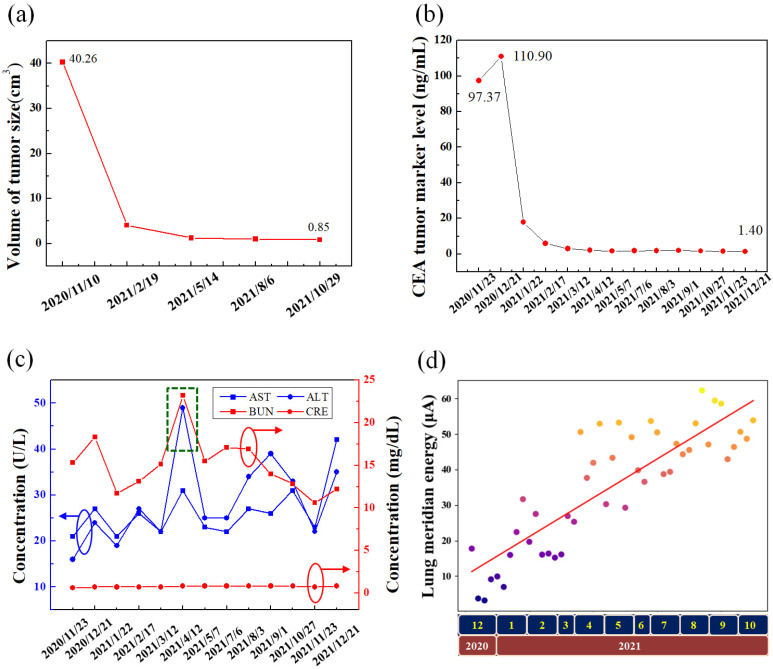
(**a**) Volume of tumor size of adenocarcinoma in situ. (**b**) Blood test for CEA marker. (**c**) Blood test of AST, ALT, BUN, and CRE. (**d**) Lung meridian energy level measurement (The measurement values at different time are represented in different color circles).

According to the response evaluation criteria for tumor size (Figure 1b,c), the CT scans display a progressive decrease in the NSCLC tumor. The patient’s clinical course is presented in Table 1. The good correlation between tumor volumes calculated with the formula π/6 × L × W × H and the actual tumor masses has been investigated [25]. After a year of combined therapy, the in situ volume of the adenocarcinoma tumor size became 47-fold smaller than the prior image on 10 November 2020 (Figure 3a). Carcinoembryonic antigen (CEA) was significantly reduced (Figure 3b). The blood tests for aspartate aminotransferase (AST), alanine aminotransferase (ALT), blood urea nitrogen (BUN), and creatinine (CRE) have also been checked for 1 year (Figure 3c).

On the other hand, the lung meridian energy was evaluated in our case based on traditional Chinese medicine. According to the Meridian Energy Analysis Device (Model: ME–100; Medpex Inc., Taichung, Taiwan), when the current on an acupoint is less than 50 μA, it represents a deficiency syndrome of the relative meridian. A good progressive increase in the lung meridian was demonstrated (Figure 3d), changing from an extremely low (deficiency) to a normal state. Currently, the patient remains clinically stable, without any treatment-associated severe adverse events.

## 3. Discussion

Previous studies have suggested that PBM may increase proliferation of cells in some malignant cell lines [26,27,28]. However, the results cannot directly conclude that PBM would aggravate the tumor cell in vivo [29] because the human immune system can also be induced by PBM [14]. In addition, the survival and recurrence rates of 41 patients with stage II and III breast cancer treated by PBM have been investigated [30]. The result showed a higher survival rate (100% in stage II and 94.44% in stage III) with PBM radiation before and after the surgery for 2 years.

In targeted therapy, ceritinib is highly selective for driver oncogenes of ALK in NSCLC. Ceritinib led to the suppression of ALK phosphorylation, as well as the downstream signaling pathways that is active against ALK-positive cancer cells [31]. However, the treatment of ceritinib caused many adverse effects (AEs). Elevated transaminase is one of the AEs associated with the oral administration of ceritinib, which was manifested by the increase in AST and ALT. In addition, ceritinib increased BUN, red rashes, and severe diarrhea. Although diarrhea can be improved with the oral administration of Loperamide HCL, there will be the side effect of dizziness. In the present study, PBM was radiated on the target or acupoints to reduce the AEs. The concentration of ASL, ALT, and BUN returned to the normal state (Figure 3c) after an 830 nm laser was radiated on BL 18 (36.58 J/cm^2^) and KI 1 (109.74 J/cm^2^) acupoints twice a day for 1 month (Figure 2b). In addition, there were red rashes on the back of the hands associated with the oral administration of ceritinib for 1 month. A 650 nm laser (8.54 J/cm^2^) was used to radiate on the target three times a day. The red rashes disappeared after a 10-day treatment. In addition, severe diarrhea stopped after the use of an 830 nm laser radiated on ST 25 acupoint (36.58 J/cm^2^), ST 36 acupoint (109.74 J/cm^2^), and stomach area (73.16 J/cm^2^) (Figure 2c), and severe dizziness stopped after an 830 nm laser (73.16 J/cm^2^) radiated on LU 11 and LU 07 acupoints (Figure 2d).

*A. cinnamomea’s* antitumor activity has been studied [32,33]. It can induce the apoptosis of human breast cancer cells [32] and effectively impede the proliferation of human NSCLC [33]. An ethanol extract of *A. cinnamomea* has also been shown to inhibit the migration of highly metastatic CL1-5 human lung adenocarcinoma cells by reducing the expression of matrix metalloproteinase-2/9 via the mitogen-activated protein kinase and phosphatidylinositiol-3–kinase/Akt signaling pathways [34]. Antcin K is the most abundant triterpenoid, and it can be extracted from *A. cinnamomea* [35]. This extract was able to inhibit the metastasis of human hepatoma cells through the suppression of integrin-mediated adhesion, migration, and invasion. On the other hand, scientists have investigated *G. lucidum’s* high antitumor activity and its pathways in cancer cells [36,37,38,39]. Polysaccharides from *G. lucidum* can increase protein kinase C, p38 mitogen-activated protein kinase, and other tyrosine kinase (Hck and Lyn) activities [36]. Ganoderan B (a glycan of *G. lucidum* fruit bodies) significantly inhibits the growth, invasion, and migration of, as well as induces apoptosis inm NSCLC cells through the extracellular signal-regulated protein kinase signaling pathway [37]. Moreover, the dose-dependence of *G. lucidum* demonstrated different inhibition rates of Sarcoma-180 cells [40]. *G. lucidum* at 5.4 g per day for 12 weeks was administered to different patients with advanced cancer [38]. The results show a series of cellular immunological enhancements, including interleukin (IL)-2, IL-6, and interferon-γ secretion in plasma and NK cell activity, but the levels of IL-1 and tumor necrosis factor-α decreased in 30 assessable patients after *G. lucidum* treatment. In addition, the same dose of *G. lucidum* was given to advanced colorectal cancer patients [39]. A similar pathway can be found in the previous study [38]. The downregulation of TNF-α and IL-1 improved cancer cachexia [41,42]. Thus, *A. cinnamomea* and *G. lucidum* show a great potential for antitumor activity. The dose of *G. lucidum* in previous studies (5.4 g per day) [38,39] was consistent with ACGL (*A.* *cinnamomea* (50%) and *G. lucidum* (50%; 450 mg/capsule × 12 capsules = 5.4 g per day) in our study.

This study shows the following integrative effects. Owing to ceritinib, PBM, and ACGL, the size of the adenocarcinoma in situ was reduced from 40.26 to 4.07 cm^3^ within 100 days. On the other hand, CEA was reduced from 97.37 to 1.40 (ng/mL) in a year.

## 4. Conclusions

In view of the supporting evidence for PBM and ACGL in cancer therapy, combined therapy was used to effectively inhibit human lung adenocarcinoma cells in this case. According to the chest CT and CEA marker, the primary lesion of the tumor responded well to combined therapy. The size of the adenocarcinoma in situ and multiple pulmonary metastases were reduced, and the CEA tumor marker level decreased rapidly. We recommend PBM and ACGL as complementary medicines for NSCLC patients. The present case may help clinicians develop a strategy for NSCLC treatment. However, more scientific evidence is needed to clarify the therapeutic strategies of PBM and ACGL.

## Figures and Tables

**Table 1:** The clinical course of the patient with combined therapy for 1 year.

Time	Image Modality	Clinical Findings	Combined Therapy
10 November 2020	Chest CT	Left upper lung cancer with lung-to-lung metastases. The size of left upper lung cancer tissue was larger than 5 cm.	ACGL and PBM therapy: (1) 6 capsules of ACGL per day; (2) 830 nm laser (30 mW, 10 Hz, 50% duty cycle) was used to radiate on the acupoints and the target (Once a day). 4 steps of 830 nm laser radiation: (i) LU11 and PC 9 acupoints; (ii) LU 11 and TE 1 acupoints; (iii) Apex of lung; (iv) BL 13 acupoint.
20 November 2020	-	-	Increase ACGL dose: 8 capsules of ACGL per day.
27 November 2020	Brain CT	No definite metastatic lesion.	
30 November 2020	-	-	Increase ACGL dose: 10 capsules of ACGL per day
18 December 2020	-	Lung adenocarcinoma confirmed diagnosis (advanced NSCLC, Stage IVa)	Ceritinib, PBM and ACGL: (3) 3 capsules of ceritinib per day (150 mg/capsule); (4) 10 capsules of ACGL per day; (5) 830 nm laser is used twice a day.
24 January 2021	-	(1) Red rash on the back of the hand (Side effect of Ceritinib). (2) Severe diarrhea (Side effect of Ceritinib).	PBM therapy: (6) Red rashes disappeared after 650 nm laser (7 mW, 10 Hz, 50% duty cycle, 10 min, 8.54 J/cm^2^) radiated on the back of the hand 3 times a day for 10 days; (7) Severe diarrhea stopped after 830 nm laser radiated on ST 25 acupoint (30 mW, 10 min, 36.58 J/cm^2^), ST 36 acupoint (30 mW, 30 min, 109.74 J/cm^2^), and stomach area (30 mW, 20 min, 73.16 J/cm^2^).
27 January 2021	-	Severe dizziness (Side effect of Loperamide HCL).	PBM therapy: Severe dizziness stopped after 830 nm laser (30 mW, 20 min, 73.16 J/cm^2^) radiated on LU 11 and LU 07 acupoints.
3 February 2021	-	Red rashes on the back of the hand were significantly improved.	
19 February 2021	Chest CT Brain CT	For chest CT: Left upper lung cancer with bilateral lung metastases is smaller than prior image on 10 November 2020. For brain CT: No definite metastatic lesion.	
16 April 2021	-	Higher concentration was found in ASL, ALT, and BUN.	PBM therapy: The concentration of ASL, ALT, and BUN decreased after 830 nm laser radiated on BL 18 acupoint (30 mW, 10 min, 36.58 J/cm^2^) and KI 1 acupoint (30 mW, 30 min, 109.74 J/cm^2^) twice a day.
12 May 2021	Brain MRI	A tiny enhancing nodule in right frontal subcortical region and another in left cerebellum.	
14 May 2021	Chest CT	Left upper lung cancer is smaller than prior image on 19 February 2021.	
3 August 2021	Brain MRI	Stable tiny enhancing nodules in right frontal subcortical region and left cerebellum compared with prior image on 12 May 2021.	Increase ACGL dose: 12 capsules of ACGL per day.
6 August 2021	Chest CT	A small decreased of left upper lung cancer with bilateral lung metastases compared with prior image on 14 May 2021.	
29 October 2021	Chest CT	No significant interval changes as compared with prior CT image on 6 August 2021	
25 November 2021	Brain MRI	Stable tiny enhancing nodules regions compared with prior image on 3 August 2021.	

LU 11: Shaoshang acupoint; PC 9: Zhongchong acupoint; TE 1: Guanchong acupoint; BL 13: Feishu acupoint; ST25: Tianshu acu-point; ST 36: Zusanli acupoint; LU 07: Lieque acupoint; BL 18: Ganshu acupoint; KI 1: Yongquan acupoint.

## Data Availability

The data used to support the findings of this study are included within the article.
